# Trigeminal neuralgia caused by cavernoma: A case report with literature review

**DOI:** 10.3389/fneur.2022.982503

**Published:** 2022-09-16

**Authors:** Hongyu Liu, Chuanbiao Chen, Yuyang Liu, Jialin Liu, Xinguang Yu, Ling Chen

**Affiliations:** ^1^Department of Neurosurgery, Hainan Hospital of Chinese PLA General Hospital, Sanya, China; ^2^Department of Neurosurgery, First Medical Center of Chinese PLA General Hospital, Beijing, China

**Keywords:** cavernoma, case report, magnetic resonance imaging, trigeminal neuralgia, trigeminal nerve

## Abstract

Cavernoma is the second most common cerebrovascular lesion. Cavernoma involving the cranial nerves is very rare. Only 15 cases of cavernoma presenting with trigeminal neuralgia (TN) have been previously reported. Here, we report a rare case of cavernoma manifesting with TN. A young female patient with a 15-day history of right-sided lancinating pain in the face, difficulty in opening the mouth, and hearing dysesthesia. Magnetic resonance imaging (MRI) revealed a well-demarcated lesion in the cerebellopontine angle related closely to the root of the trigeminal nerve. The initial impression was that of a neurinoma. The lesion was surgically resected *via* the retrosigmoid approach, postoperative pathological analysis confirmed the diagnosis of cavernoma, and the patient's pain and difficulty in opening the mouth resolved completely. We presented the 16^th^ documented case of cavernoma with TN. Although cavernoma involving the trigeminal nerve is extremely rare, this diagnosis should be taken into consideration when a lesion in the cerebellopontine angle is detected on MRI, and the clinical manifestation is consistent with that of secondary TN. Specialized MRI sequences, such as susceptibility weighted imaging (SWI), gradient echo T2, and constructive interference in steady-state (CISS)-weighted imaging, aid in establishing the diagnosis. Resection *via* craniotomy may be the primary management strategy for cavernoma causing TN. In addition, gamma knife radiosurgery (GKRS) and percutaneous balloon compression (PBC) may ameliorate the pain to some extent.

## Introduction

Cavernoma, also known as cavernous malformation, cavernous angioma, or cavernous haemangioma, is the second most common cerebrovascular lesion, with a reported incidence of 0.5% (1, 2). About 40% of cavernoma are asymptomatic, and the typical clinical manifestations include epilepsy, focal deficits, headaches, and intracranial hemorrhage (1, 2). Cavernoma can occur anywhere along the neuraxis; however, cavernoma involving the cranial nerves is very rare (3). The causes of trigeminal neuralgia (TN) include microvascular compression and compression due to space-occupying lesions, multiple sclerosis, and herpes zoster (4). Only 15 cases of cavernoma presenting with TN have been previously reported (3, 5–18). Here, we report a rare case of cavernoma manifesting with TN.

## Case description

**Figure 1:**
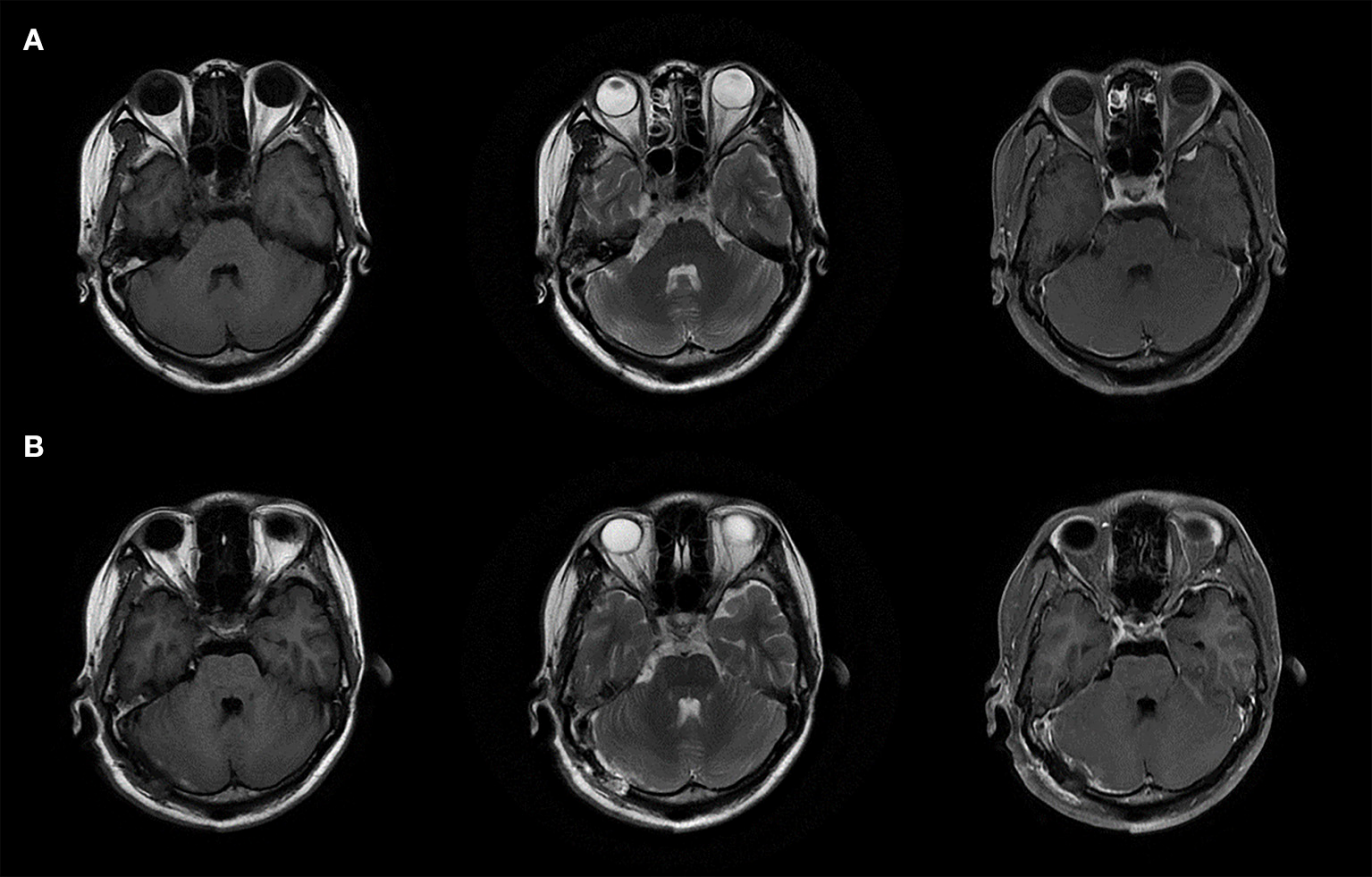
MRI images of the lesion. **(A)** Preoperative brain MRI images. **(B)** Postoperative brain MRI images.

**Figure 2:**
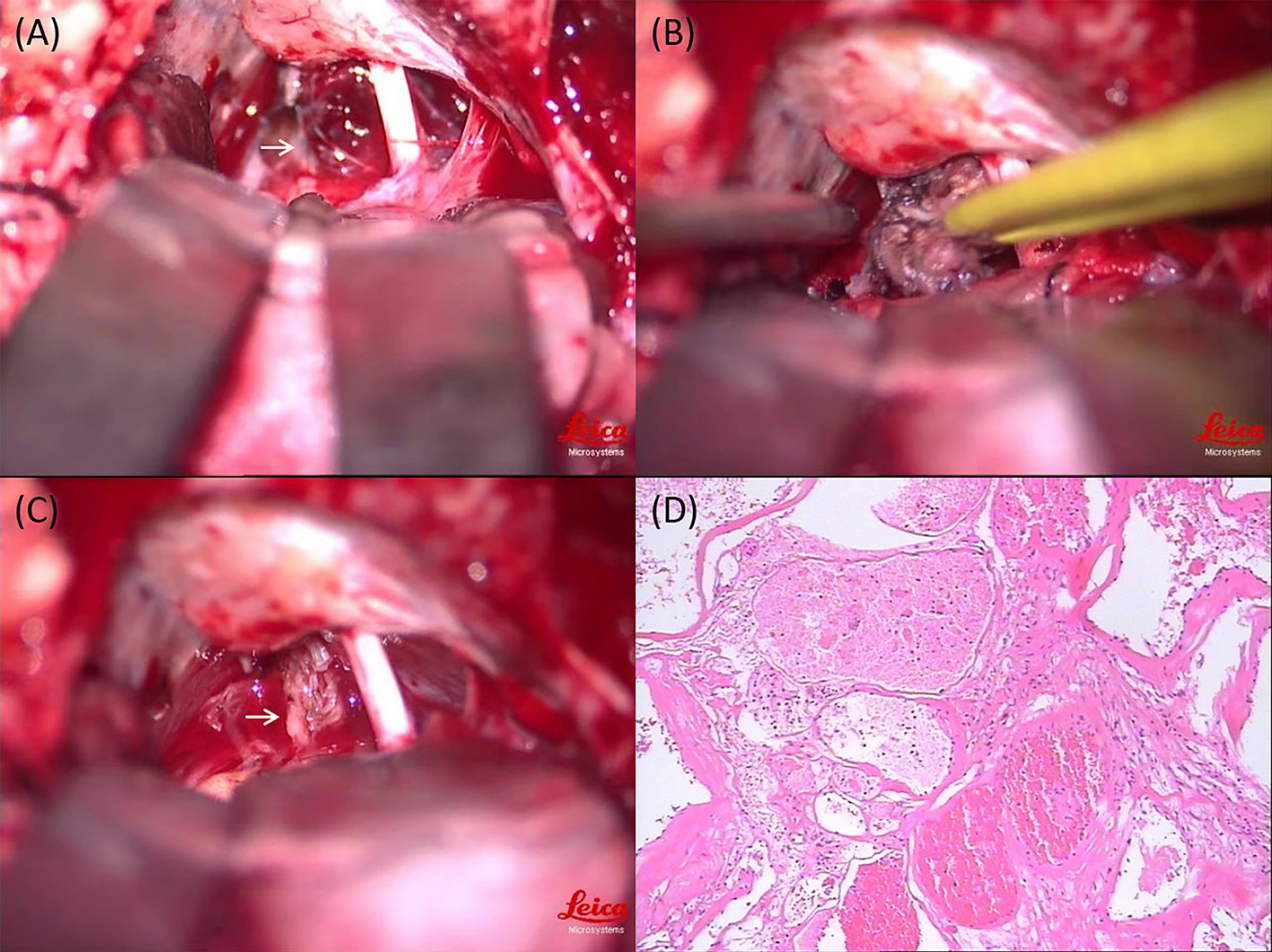
Intraoperative photography and histopathological examination of the lesion. **(A)** Intraoperative view: the lesion was dark-red, had an abundant blood supply, and contained vessel-like structures (white arrow). **(B)** Intraoperative view: the lesion was resected en bloc. **(C)** Intraoperative view: the right trigeminal nerve motor root was carefully preserved (white arrow). **(D)** Histopathological examination of the lesion (magnification × 100).

A 29-year-old woman with no past medical history presented with a 15-day history of paroxysmal lancinating right facial pain radiating to the jaw, in addition to a difficulty in opening the mouth. Pain attacks were often triggered by chewing. The facial pain lasted for few minutes and occurred three to five times per day. Treatment with carbamazepine was ineffective. Neurological examination revealed mild hearing loss on the right side. Her corneal reflex and facial sensation were normal. Muscle strength and sensation of limbs were normal. No ataxia or pathological reflex was observed. Preoperative brain magnetic resonance imaging (MRI) showed a well-demarcated, oval mass measuring 17 × 11 × 10 mm in the right cerebellopontine angle (Figure 1A). The lesion was closely related to the right trigeminal nerve and appeared slightly hypointense on T1-weighted sequence, slightly hyperintense on T2-weighted sequence, and non-enhanced on T1 contrast sequence. Surgical resection was performed *via* the right retrosigmoid approach. The lesion was dark-red, had an abundant blood supply, and contained vessel-like structures (Figure 2A). In order to avoid stretch injury of posterior cranial nerves and excessive traction of right cerebellar hemisphere which could lead to cerebellar contusion and postoperative edema, the lateral part of pons besides the lesion was not exposed. Eventually, the lesion was resected en bloc (Figure 2B). Sensory root of the right trigeminal nerve could not be preserved because of the obscure boundary between it and the lesion. However, the right trigeminal nerve motor root was carefully preserved (Figure 2C). Following surgery, pain and difficulty in opening the mouth disappeared immediately, but mild hearing loss did not improve. In addition, the patient developed right facial hypoesthesia.

The pathological diagnosis was cavernoma. Microscopically, malformed vascular tissue and hyaline degeneration of the vascular wall were observed (Figure 2D). Postoperative brain MRI showed that the tumor had been completely removed (Figure 1B), and the patient was discharged on the 18^th^ postoperative day. There was no recurrence of pain during the 3-year follow-up period. Her postoperative facial hypoesthesia on the right side was not improved.

## Discussion

TN is the most common form of craniofacial neuropathic pain, which can be classified into 3 types, idiopathic, classic, and secondary TN (19). Idiopathic TN is characterized by unapparent causes. Classic TN, the most frequent type clinically, is caused by neurovascular compression in the trigeminal nerve root (19). Microvascular decompression is a particularly effective treatment for classic TN (20). Secondary TN is generally triggered by a major neurologic disease such as multiple sclerosis or the growth of cerebellopontine angle tumors. Tumors that cause TN include meningiomas, acoustic neuromas, epidermoid cysts, and cholesteatomas. These tumors are benign and compress trigeminal root entry zone (19). In both classic and secondary TN, the foremost mechanism is focal demyelination of primary afferents near the trigeminal root entry zone caused by local compression, which may trigger paroxysmal ectopic discharges. In addition, infiltrative tumors could lead to axonal degeneration (19). Zhong (20) proposed that aging would lead cranial nerve root and surrounding vessel get closer mutually and finally the neurovascular conflict happened. The nerve incurs demyelination due to interfacial friction associated with pulse. Once pressure stimulation occur, an impulse or excitability may generate from the axon cranial nerve root. Voltage-gated ion channels and mechanosensitive ion channels are activated subsequently. Thus, they serve as a trigger for a painful attack by ectopic action potential. During the process, inflammation factors like TNF-α and IL-6 may play an indispensable role in mediating the development of transmembrane ion channels (21). This hypothesis could elucidate the clinical phenomenon that secondary TN cases were mostly caused by meningioma or cholesteatoma instead of schwannoma, because the latter could insulate the nerve by providing proliferative sheaths (21–23).

Cavernoma with TN occurs extremely rare. Fehlings et al. (5) reported the first case in 1988, and thus far, only 16 cases, including our case, have been reported (Table 1) (3, 4, 6–18). Among these, 11 were treated with craniotomy, two with pharmacotherapy, one with gamma knife radiosurgery (GKRS), and one with percutaneous balloon compression (PBC); while one case was managed conservatively. Resection *via* craniotomy is the primary management strategy, and most patients have good outcomes after surgery (16–18). Complete resection of cavernoma located in the brainstem is crucial, since incomplete resection could increase the re-bleeding risk by up to 43% (13). In addition, medication and other less invasive treatments including GKRS and PBC can be considered if risk of open surgery is high (8, 9, 16, 18).

**Table 1:** Reported cases of Cavernous malformation with Trigeminal neuralgia.

**References**	**Patient**	**Treatment**	**Efficacy of pain relief according to Brisman's criteria**
Fehlings et al. (5)	33 Male	Craniotomy	Cure
Saito et al. (6)	45 Female	Craniotomy	Cure
De Benedittis (7)	62 Male	Craniotomy	Death
Shimpo (8)	67 Male	Pharmacotherapy (carbamazepine)	Effectiveness
Vitek and Tettenborn (9)	61 Male	Pharmacotherapy (gabapentin)	Obvious effectiveness
Deshmukh et al. (3)	52 Female	Craniotomy	Cure
Mascarenhas et al. (10)	54 Male	Craniotomy	Cure
Stellmann et al. (11)	55 Female	Spontaneously disappeared	Cure
Seckin et al. (12)	56 Male	Craniotomy	Cure
Cenzato et al. (13)	45 Male	Craniotomy	Cure
Adachi et al. (14)	62 Male	Craniotomy	Cure
Frossard et al. (15)	56 Female	Craniotomy	Not reported
Pease et al. (16)	80 Female	GKRS	Obvious effectiveness
Scavo et al. (17)	62 Female	Craniotomy	Cure
Zhang et al. (18)	37 Male	PBC	Cure
Present Study	29 Female	Craniotomy	Cure

GKRS, gamma knife radiosurgery; PBC, percutaneous balloon compression.

In 2014, Adachi et al. (14) reviewed 11 cases and classified them into 4 types according to the origin of cavernoma as follows: type G (within the Gasserian ganglion); type C (between the cisternal and intra-axial portions of the trigeminal nerve root); type P (within the intra-axial trigeminal nerve root in the pons); and type S (within the spinal tract of the trigeminal nerve root). This classification helps in the selection of treatment modality and surgical approach. For instance, five patients with type G or C cavernoma were all treated with surgery without complications, and their pain was relieved. However, the surgical risk for type P and S was reported to be higher than that for other types (14). One patient with type P cavernoma died of postoperative hemorrhage (7). Therefore, pharmacotherapy could be considered for these two types, and carbamazepine and gabapentin are effective for relieving pain (8, 9). In one case, pain of a patient with type S cavernoma disappeared spontaneously (11). Surgical risk was acceptable in our case as it was type C, and resection *via* the retrosigmoid approach that can expose the lesion sufficiently is the most appropriate strategy for such cases.

Stereotactic radiotherapy is an effective treatment for cavernoma with TN, especially for patients who cannot tolerate surgery. Pease et al. reported the first case of an elderly patient treated with GKRS. After irradiation of the trigeminal nerve, the pain was alleviated significantly without major complications, which confirmed the safety and effectiveness of GKRS (16).

PBC, a traditional therapy for TN and not for cavernoma, is also effective for pain relief. Zhang et al. reported the first case of using PBC to treat TN caused by cavernoma. Considering the surgical risk, the patient refused resection *via* craniotomy. After PBC of the Gasserian ganglion, the patients'pain disappeared completely, which demonstrated that PBC is a reasonable treatment for patients with cavernoma and TN who are reluctant to undergo craniotomy (18).

Cavernoma is most commonly diagnosed using MRI, and it typically appears as a heterogeneous lesion surrounded by a hypointense hemosiderin rim (17). However, preoperative differential diagnosis may be difficult because of atypical imaging findings (10, 12, 15). For example, the most likely preoperative diagnosis in our case was trigeminal neurinoma due to the close relationship between the lesion and right trigeminal nerve observed on MRI, and the signal intensity of the lesion on T1-weighted, T2-weighted, and T1 contrast sequences. The typical symptoms of secondary TN and neurological examination also supported this diagnosis. Susceptibility weighted imaging (SWI) and gradient echo T2 sequence are better able to detect the impressive hemosiderin deposition in the lesion (15, 17, 18). Adachi et al. (14) showed that vague hemosiderin rim and developmental venous anomaly could be observed on constructive interference in steady-state (CISS)-weighted imaging that was helpful in delineating the intracisternal segment of the trigeminal nerve (24). Therefore, specialized MRI sequences may be effective for diagnosing cavernoma with TN.

## Conclusions

We presented the 16^th^ documented case of cavernoma with TN. Although cavernoma involving the trigeminal nerve is extremely rare, this diagnosis should be taken into consideration when a lesion in the cerebellopontine angle is detected on MRI, and the clinical manifestation is consistent with that of secondary TN. Specialized MRI sequences, such as SWI, gradient echo T2, and CISS-weighted imaging, aid in establishing the diagnosis. Resection *via* craniotomy may be the primary management strategy for cavernoma causing TN. In addition, GKRS and PBC may ameliorate the pain to some extent.

## Data availability statement

The original contributions presented in the study are included in the article/supplementary material, further inquiries can be directed to the corresponding authors.

## Ethics statement

Written informed consent was obtained from the individual(s) for the publication of any potentially identifiable images or data included in this article.

## Author contributions

HL, CC, and YL collected the data and prepared the manuscript. JL analyzed the data and created the tables and figures. XY and LC designed and supervised the work. All authors agree to be accountable for the content of the work, contributed to the article, and approved the submitted version.

## Funding

This work was supported by National Natural Science Foundation of China, Grant/Award Number: 82172680.

## Conflict of interest

The authors declare that the research was conducted in the absence of any commercial or financial relationships that could be construed as a potential conflict of interest.

## Publisher's note

All claims expressed in this article are solely those of the authors and do not necessarily represent those of their affiliated organizations, or those of the publisher, the editors and the reviewers. Any product that may be evaluated in this article, or claim that may be made by its manufacturer, is not guaranteed or endorsed by the publisher.

## Supplementary material

The Supplementary Material for this article can be found online at: https://www.frontiersin.org/articles/10.3389/fneur.2022.982503/full#supplementary-material
